# Independent and combined associations of dietary antioxidant exposure with all-cause and cause-specific mortality in the general population

**DOI:** 10.1017/jns.2026.10117

**Published:** 2026-07-07

**Authors:** Zhijun Liu, Zhuojian He, Yanfei Xing

**Affiliations:** 1 Department of Child Health Care, Guangzhou Women and Children’s Medical Center, Guangzhou Medical University, China; 2 School of public health, Guangzhou Medical University, China; 3 Department of Clinical Medicine, The First Clinical School, Guangzhou Medical University, China

**Keywords:** All-cause mortality, Cause-specific mortality, CDAI, Diet antioxidants, NHANES

## Abstract

This study aimed to determine if habitual intake of key dietary antioxidants, both individually and collectively, is associated with all-cause and cause-specific mortality among U.S. adults, given that oxidative stress heightens cardiovascular and cancer mortality risk. This prospective cohort study analysed 34,955 participants from the National Health and Nutrition Examination Survey (NHANES) 2003–2018. Intakes of vitamins A, C, E, zinc, selenium, and carotenoids were assessed, and a composite dietary antioxidant index (CDAI) was calculated. The principal results, from 347,580 person-years of follow-up where 4,456 deaths occurred (1,368 CVD; 1,038 cancer), showed significant associations. Compared with the lowest intake quintile, the highest quintile of vitamin E was associated with lower all-cause mortality (HR: 0.69, 95% CI: 0.58–0.83) and CVD mortality (HR: 0.67, 0.48–0.92). High carotenoid intake was inversely associated with all-cause mortality (HR: 0.77, 0.67–0.88). Participants in the highest CDAI quintile experienced an 18% lower risk of all-cause mortality (HR: 0.82, 0.70–0.97) and a 39% lower risk of cancer mortality (HR: 0.61, 0.45–0.84). Dose–response relationships for vitamins A, C, selenium, and zinc were U-or L-shaped, and WQS analyses assigned the greatest weights to vitamin A or C. In conclusion, while individual antioxidants like vitamin E show strong protective associations, the evidence collectively suggests that greater overall antioxidant exposure from a varied diet is linked to materially lower risks of death. This reinforces that focusing on diets rich in diverse antioxidant sources is superior to single-nutrient strategies.

## Introduction

The prevalence of cardiovascular diseases (CVD)^(1)^ and cancer^(2)^ continues to rise and has become a major challenge to global public health. In 2019, CVD caused approximately 18.6 million deaths globally and accounted for nearly 32% of all deaths each year.^(3)^ In 2022, there were nearly 20 million new cancer cases globally and 9.7 million cancer-related deaths.^(4)^

A critical element in the development of CVDs and cancers is oxidative stress, which involves excessive reactive oxygen species (ROS) and a disturbance in antioxidant homeostasis. This elevated oxidative stress leads to DNA damage and gene mutations,^(5)^ facilitates cellular deterioration and injury, and contributes to endothelial dysfunction and vascular remodelling.^(6)^ Elevated levels of oxidative stress exacerbate inflammation, which is associated with a higher risk of cancer and cardiovascular disease.^(7,8)^

As an inexpensive and simple non-pharmacological strategy, dietary treatments have attracted a lot of attention.^(9)^ Food-based antioxidants might decrease oxidative stress, potentially reducing the risk of cancer and heart disease. Research has shown that antioxidants found in the diet, such as selenium, carotenoids, zinc, and vitamins A, C, and E, are linked to a lower risk of CVD and cancer mortality.^(10–12)^

Although observational research indicates that dietary antioxidants might lower mortality risk, the evidence remains inconsistent and is often limited to individual nutrients or specific populations.^(13–18)^ Aggregating intake data for key antioxidant micronutrients — including vitamin A, vitamin C, vitamin E, selenium, zinc, and assorted carotenoids — the Composite Index of Dietary Antioxidants (CDAI) serves as a population-wide proxy for total antioxidant consumption. Higher CDAI levels have consistently been linked to reductions in both overall and cause-specific mortality.^(19)^ In some cohorts, a greater intake of dietary antioxidants was also associated with a reduction in mortality, but the shape of this relationship — whether it is linear or U-shaped — remains uncertain. Furthermore, the relative contribution of each antioxidant nutrient in the dietary matrix has not been clarified.

Given the widespread use of supplements and increasing public interest in antioxidant-rich diets, clarifying the association between individual and combined antioxidant intake and mortality is essential. This study aimed to assess the association of independent intake of dietary antioxidants, as well as total dietary antioxidant intake as measured by the CDAI, with all-cause and cause-specific mortality in a nationally representative U.S. population.

## Methods

### Study population

**Figure 1.**
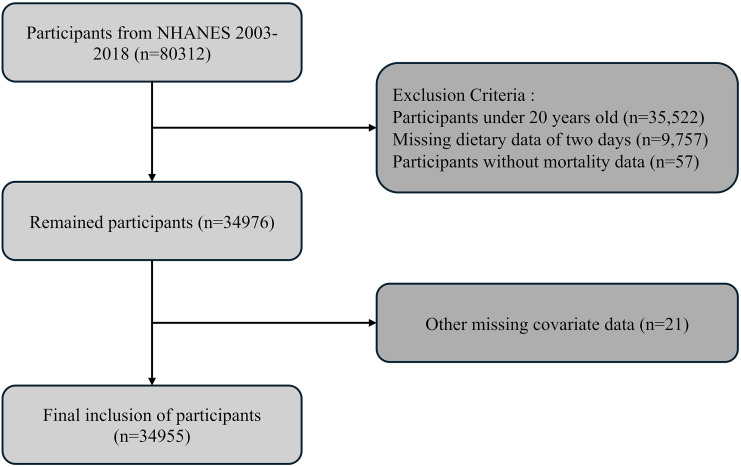
Figure 1 long description. Participants screening flow chart.

The analytic sample comprised respondents from eight successive NHANES cycles (2003–2018), yielding a nationally representative cohort of U.S. adults. Initially, 80,312 participants were identified. Those aged under 20 years (*n* = 35,522), with incomplete dietary recall data (*n* = 9,757), missing survival follow-up information (*n* = 57) were excluded. A hybrid approach was employed to handle missing data. Multiple imputation was utilised to resolve missing values exclusively for continuous covariates to maximise statistical power. However, a minimal number of participants (*n* = 21, approximately 0.06% of the eligible cohort) with missing categorical covariate data (specifically, smoking status) were not imputed and were directly excluded from the analyses. Given the extremely low missing rate, this specific exclusion avoids potential imputation bias without compromising statistical validity. The final study cohort consisted of 34,955 participants. The criteria for participant inclusion and exclusion are detailed in a diagram shown in Figure 1.

### Exposure variable

Dietary antioxidant intake was estimated from the average of two non-consecutive 24-hour dietary recalls. To minimise recall bias, NHANES utilises the USDA Automated Multiple-Pass Method (AMPM), a validated, fully computerised five-step interview process.^(20)^ The initial recall was administered face-to-face at the mobile examination centre using standard measuring guides for portion size estimation, followed by a telephone interview 3 to 10 days later. Nutrient estimates were derived from the USDA Food and Nutrient Database for Dietary Studies (FNDDS). The analysis considered six principal antioxidant nutrients — vitamin A, vitamin C, vitamin E, zinc, selenium, and a pooled group of carotenoids. Total antioxidant exposure was estimated with an adapted CDAI, calculated by z-scoring each nutrient intake and adding the resulting standardised values.^(21)^

$$\mathrm{CDAI}=\sum_{i=1}^{n=6}\frac{\mathrm{Diet}\mathrm{intake}-\mathrm{Mean}}{\mathrm{SD}}$$

### Assessment of mortality

Using NCHS algorithms, mortality data were linked from NHANES to the National Death Index, covering up to the end of December 2019. The study’s main endpoints were mortality rates from all causes, cardiovascular diseases (CVD), and cancer. Death causes were determined using ICD-10 codes. All-cause mortality included deaths from any reason, such as heart diseases (I00-I09, I11, I13, I20-I51), cancers (C00-C97), accidents (V01-X59, Y85-Y86), cerebrovascular diseases (I60-I69), diabetes (E10-E14), and others. Cancer deaths were identified by codes C00-C97. CVD mortality was defined as deaths due to major cardiovascular diseases and cerebrovascular diseases (codes: I00-I09, I11, I13, I20-I51, and I60-I69). The period of follow-up was from enrolment to December 31, 2019, or until death.

### Covariates

The study utilised covariates spanning demographic characteristics (age, sex, race, poverty income ratio (PIR), weight status), lifestyle factors (physical activity (PA), smoking status, alcohol consumption, total dietary energy intake), and health conditions (hypertension, diabetes, cancer, cardiovascular disease). Weight status was grouped as normal (<25 kg/m^2^), overweight (25–30 kg/m^2^), or obese (>30 kg/m^2^), and PIR as low (<1.0), middle (1.0–3.0), or high income (≥3.0). Alcohol consumption was classified as never/light or heavy drinking, while smoking status included current (>100 lifetime cigarettes), former (>100 cigarettes and quit), or non-smokers. Physical activity levels were categorised as no, moderate, intense, or moderate + intense. Health conditions were defined clinically: diabetes (self-reported, medication, glycohemoglobin ≥ 6.5%, or fasting blood glucose ≥ 126 mg/dL), hypertension (self-reported, medication, or blood pressure ≥ 140/90 mmHg), and self-reported cardiovascular disease or cancer. These covariates established a robust basis for the subsequent statistical analysis.

### Statistical analysis

Given NHANES’s multistage, probability-based sampling scheme, all analyses applied the appropriate survey weights. The CDAI was stratified into quintiles (Q1–Q5). Continuous variables with non-normal distributions were described as the median and interquartile range [M (IQR)], and between-group differences were assessed using the Kruskal–Wallis test. Categorical variables were described as frequencies and percentages [n (%)], and between-group differences were assessed using the chi-square test. The crude model was unadjusted. Model 1 controlled for gender, age, race, weight status, and PIR. Model 2 added smoking status, alcohol use, physical activity, and total energy intake, while Model 3 incorporated additional clinical factors: hypertension, diabetes, cancer (unadjusted for cancer mortality), and CVD (unadjusted for CVD mortality). The relationship between antioxidant levels and the CDAI across various mortality categories was elucidated using the Restricted Cubic Spline (RCS) model. Differences in CDAI across groups classified by survival, all-cause mortality, CVD mortality, and cancer mortality were found using the Kruskal–Wallis test. To evaluate the impact of dietary antioxidants on mortality, we employed weighted quantile sum (WQS) regression. Individual weights and overall effects were calculated for the six antioxidants, with an overall weight of up to one.

Subgroup analyses based on gender, age, race, and alcohol consumption were used to evaluate the impact of CDAI on all-cause and cause-specific mortality. The survival probability for each CDAI quintile (Q1–Q5) was displayed using Kaplan–Meier curves. The analyses were performed using R (v4.1.1), with statistical significance determined by a two-sided *p*-value <0.05.

## Results

### Baseline characteristics

Descriptive characteristics of participants by CDAI quintile are shown in Table 1. In the NHANES cohort, there were 34,955 participants with a median age of 47.00 (IQR:33.00, 60.00) years (52.3% female, 68.05% non-Hispanic White). Higher CDAI levels (Q5) were more frequently observed among individuals who were younger, male, more physically active, had higher incomes (PIR ≥ 3.0) and greater energy intake, were more likely to be current smokers, consumed never/light amounts of alcohol, and did not have a baseline history of hypertension, diabetes, CVD, or cancer (Table 1).

**Table 1.** Baseline characteristics Table 1 long description.

	Total (*n* = 34955)	Q1 (*n* = 6991)	Q2 (*n* = 6991)	Q3 (*n* = 6991)	Q4 (*n* = 6991)	Q5 (*n* = 6991)	*P*
Age^&^, median [IQR]	47.00 (33.00, 60.00)	47.00 (32.00, 61.00)	48.00 (33.00, 62.00)	47.00 (33.00, 61.00)	47.00 (33.00, 60.00)	45.00 (32.00, 58.00)	<0.001
Energy^&^, median [IQR]	1969.50 (1523.00, 2539.50)	1280.50 (1027.50, 1583.50)	1691.50 (1411.00, 2010.00)	1950.00 (1624.00, 2309.50)	2270.50 (1895.50, 2697.50)	2780.00 (2239.50, 3387.00)	<0.001
Gender^#^, *n*(%)							<0.001
Male	16626 (47.70)	2298 (29.89)	2719 (37.08)	3229 (43.68)	3775 (54.41)	4605 (67.75)	
Female	18329 (52.30)	4693 (70.11)	4272 (62.92)	3762 (56.32)	3216 (45.59)	2386 (32.25)	
Race^#^, *n*(%)							<0.001
Mexican American	5483 (8.46)	1093 (8.32)	1178 (9.05)	1093 (8.00)	1067 (8.43)	1052 (8.53)	
Other Hispanic	2984 (5.05)	712 (6.15)	584 (4.83)	600 (5.21)	568 (4.80)	520 (4.48)	
Non-Hispanic White	15812 (68.05)	2801 (63.29)	3062 (66.49)	3211 (68.18)	3397 (70.34)	3341 (70.76)	
Non-Hispanic Black	7463 (11.33)	1885 (15.98)	1566 (12.51)	1419 (11.23)	1277 (9.15)	1316 (8.89)	
Other Race	3213 (7.11)	500 (6.27)	601 (7.12)	668 (7.38)	682 (7.28)	762 (7.34)	
Smoking status^#^, *n*(%)							<0.001
Non-smokers	6947 (20.07)	1877 (29.99)	1432 (22.61)	1220 (17.78)	1200 (16.42)	1218 (15.79)	
Former smokers	8830 (24.93)	1539 (20.21)	1702 (22.73)	1804 (25.02)	1907 (28.08)	1878 (27.37)	
Current smokers	19178 (55.00)	3575 (49.80)	3857 (54.66)	3967 (57.21)	3884 (55.50)	3895 (56.85)	
Alcohol use^#^, *n*(%)							0.096
Never/Light	23979 (64.79)	4860 (62.73)	4861 (65.17)	4773 (64.26)	4794 (65.28)	4691 (66.06)	
Heavy	10976 (35.21)	2131 (37.27)	2130 (34.83)	2218 (35.74)	2197 (34.72)	2300 (33.94)	
Weight Status^#^, *n*(%)							<0.001
Normal	9872 (30.07)	1923 (29.64)	1815 (28.91)	1940 (30.09)	2010 (29.48)	2184 (31.94)	
Overweight	13477 (37.10)	2877 (39.88)	2840 (38.81)	2758 (38.29)	2606 (36.73)	2396 (32.75)	
Obesity	11606 (32.83)	2191 (30.49)	2336 (32.28)	2293 (31.63)	2375 (33.79)	2411 (35.31)	
Hypertension^#^, *n*(%)							<0.001
No	17469 (55.83)	3115 (53.05)	3274 (52.49)	3527 (56.12)	3671 (56.91)	3882 (59.52)	
Yes	17486 (44.17)	3876 (46.95)	3717 (47.51)	3464 (43.88)	3320 (43.09)	3109 (40.48)	
Diabetes^#^, *n*(%)							0.001
No	29789 (88.83)	5814 (88.08)	5838 (87.10)	5990 (89.18)	6047 (89.28)	6100 (90.15)	
Yes	5166 (11.17)	1177 (11.92)	1153 (12.90)	1001 (10.82)	944 (10.72)	891 (9.85)	
Family poverty income ratio^#^, *n*(%)							<0.001
<1	10529 (21.53)	2778 (30.59)	2240 (24.23)	1987 (20.24)	1793 (17.38)	1731 (17.3)	
1∼3	11250 (29.30)	2333 (32.43)	2371 (31.53)	2242 (29.27)	2174 (27.73)	2130 (26.49)	
≥3	13176 (49.17)	1880 (36.98)	2380 (44.24)	2762 (50.50)	3024 (54.89)	3130 (56.15)	
Physical activity^#^, *n*(%)							<0.001
No	19055 (48.99)	4240 (53.89)	4069 (51.63)	3761 (48.47)	3655 (49.10)	3330 (43.32)	
Moderate	10139 (32.89)	1747 (29.75)	1933 (32.64)	2103 (33.52)	2098 (32.17)	2258 (35.69)	
Intense	1655 (4.73)	305 (4.54)	286 (4.03)	356 (5.09)	328 (4.30)	380 (5.55)	
Moderate +Intense	4106 (13.38)	699 (11.82)	703 (11.71)	771 (12.91)	910 (14.43)	1023 (15.44)	
CVD^#^, *n*(%)							<0.001
No	30961 (91.22)	5958 (88.86)	6089 (90.51)	6226 (90.23)	6279 (92.09)	6409 (93.72)	
Yes	3994 (8.78)	1033 (11.14)	902 (9.49)	765 (9.77)	712 (7.91)	582 (6.28)	
Cancer^#^, *n*(%)							0.093
No	31472 (89.83)	6297 (90.00)	6275 (89.16)	6271 (89.11)	6270 (89.78)	6359 (90.98)	
Yes	3483 (10.17)	694 (10.00)	716 (10.84)	720 (10.89)	721 (10.22)	632 (9.02)	

& : Kruskal–Wallis test.

# : Chi-square test.

### Relationship between antioxidant intake levels and mortality

4,456 fatalities (12.75%) occurred throughout the 34,955-person follow-up period, with 1,368 (3.91%) attributable to CVD, 1,038 (2.97%) to cancer, and 2,050 (5.86%) to other causes. The relationship between the population survivor group and the different cause of death group, with differences in the distribution of dietary antioxidant levels, is shown in supplementary Figure 2. In the population, the all-cause death and cause-specific death groups had significantly lower levels of dietary antioxidant intake, including vitamin E, selenium, zinc, and carotenoids, compared to the survivor group (Supplementary Figure 2). In Model 3, compared with Q1, the highest quintiles (Q5) of vitamin E and carotenoids were significantly associated with lower all-cause mortality, with HRs of 0.69 (95% CI: 0.58–0.83) and 0.77 (95% CI: 0.67–0.88), respectively (*P* for trend < 0.001). Reduced risks of all-cause mortality were also observed for vitamin A (Q4 HR: 0.81, 95% CI: 0.69–0.96), vitamin C (Q4 HR: 0.85, 95% CI: 0.75–0.96), and zinc (Q2 HR: 0.85, 95% CI: 0.74–0.97; Q3 HR: 0.86, 95% CI: 0.75–0.99). Regarding cancer mortality, significant inverse associations were observed for vitamin E (Q5 HR: 0.67, 95% CI: 0.46–0.96; *P* for trend = 0.007) and vitamin C (Q4 HR: 0.70, 95% CI: 0.53–0.93). For CVD mortality, vitamin E demonstrated a significant protective association (Q2 HR: 0.80, 95% CI: 0.65–0.99; Q5 HR: 0.67, 95% CI: 0.48–0.92; *P* for trend = 0.043) (Table 2).

**Figure 2.**
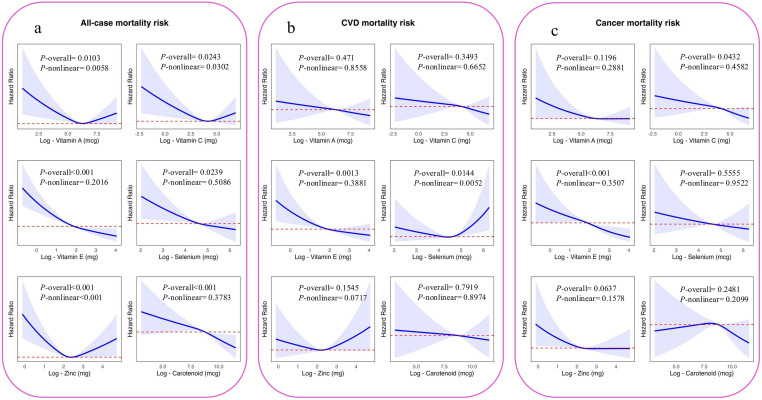
Figure 2 long description. Restricted cubic spline curve for the association of dietary antioxidants with all-cause and cause-specific mortality. (a) All-cause mortality risk; (b) CVD mortality risk; (c) cancer mortality risk.

**Table 2.** The association between dietary intake of antioxidant and all-cause mortality and cause-specific mortality Table 2 long description.

Outcomes	Variable types	Death/person	Model crude	Model 1	Model 2	Model 3
			HR (95%CI)	HR (95%CI)	HR (95%CI)	HR (95%CI)
All-cause mortality	Vitamin A	4456/34955	1.04 (1.01–1.08)	0.96 (0.92–1.01)	1.00 (0.97–1.04)	1.01 (0.97–1.05)
	Quintile					
	Q1	864/6987	1.00 (Reference)	1.00 (Reference)	1.00 (Reference)	1.00 (Reference)
	Q2	923/6993	0.96 (0.82–1.13)	0.81 (0.70–0.94)	0.88 (0.75–1.03)	0.88 (0.76–1.02)
	Q3	914/6995	1.06 (0.92–1.22)	0.81 (0.70–0.93)	0.92 (0.79–1.07)	0.90 (0.79–1.04)
	Q4	923/6992	1.02 (0.88–1.18)	0.70 (0.59–0.82)	0.81 (0.69–0.97)	0.81 (0.69–0.96)
	Q5	832/6988	1.14 (0.98–1.32)	0.81 (0.70–0.93)	0.99 (0.84–1.17)	0.98 (0.83–1.15)
	P for trend		0.055	0.045	0.650	0.745
	Vitamin E	4456/34955	0.77 (0.72–0.82)	0.85 (0.79–0.91)	0.89 (0.83–0.96)	0.88 (0.82–0.95)
	Quintile					
	Q1	1282/7001	1.00 (Reference)	1.00 (Reference)	1.00 (Reference)	1.00 (Reference)
	Q2	1019/6982	0.79 (0.70–0.89)	0.86 (0.76–0.97)	0.88 (0.77–0.99)	0.88 (0.77–1.01)
	Q3	872/7014	0.73 (0.64–0.83)	0.81 (0.70–0.93)	0.86 (0.74–0.99)	0.85 (0.73–0.99)
	Q4	708/6972	0.58 (0.50–0.66)	0.67 (0.57–0.78)	0.70 (0.59–0.83)	0.70 (0.59–0.83)
	Q5	575/6986	0.50 (0.43–0.58)	0.65 (0.55–0.76)	0.71 (0.59–0.84)	0.69 (0.58–0.83)
	P for trend		< .001	< .001	< .001	< .001
	Vitamin C	4456/34955	0.95 (0.91–0.99)	0.93 (0.89–0.99)	1.00 (0.95–1.05)	1.00 (0.95–1.05)
	Quintile					
	Q1	1086/6990	1.00 (Reference)	1.00 (Reference)	1.00 (Reference)	1.00 (Reference)
	Q2	957/6993	0.99 (0.87–1.13)	0.85 (0.74–0.98)	0.91 (0.80–1.05)	0.90 (0.79–1.03)
	Q3	873/7001	1.01 (0.87–1.16)	0.78 (0.69–0.88)	0.91 (0.80–1.03)	0.89 (0.79–1.01)
	Q4	807/6985	1.03 (0.89–1.19)	0.75 (0.66–0.84)	0.86 (0.76–0.98)	0.85 (0.75–0.96)
	Q5	733/6986	0.90 (0.79–1.04)	0.77 (0.68–0.88)	0.94 (0.83–1.07)	0.94 (0.82–1.07)
	P for trend		0.183	0.001	0.565	0.573
	Zinc	4456/34955	0.86 (0.78–0.95)	0.98 (0.93–1.04)	1.02 (0.99–1.05)	1.02 (0.99–1.05)
	Quintile					
	Q1	837/6983	1.00 (Reference)	1.00 (Reference)	1.00 (Reference)	1.00 (Reference)
	Q2	806/6995	0.76 (0.67–0.87)	0.84 (0.74–0.95)	0.88 (0.77–1.01)	0.85 (0.74–0.97)
	Q3	922/7007	0.68 (0.59–0.78)	0.82 (0.72–0.94)	0.90 (0.78–1.04)	0.86 (0.75–0.99)
	Q4	910/6982	0.62 (0.54–0.72)	0.80 (0.69–0.93)	0.90 (0.75–1.08)	0.86 (0.72–1.03)
	Q5	981/6988	0.55 (0.47–0.64)	0.80 (0.67–0.95)	0.94 (0.77–1.16)	0.89 (0.73–1.08)
	P for trend		<.001	0.024	0.908	0.551
	Selenium	4456/34955	0.71 (0.68–0.75)	0.90 (0.85–0.96)	0.95 (0.88–1.03)	0.94 (0.88–1.02)
	Quintile					
	Q1	1248/6988	1.00 (Reference)	1.00 (Reference)	1.00 (Reference)	1.00 (Reference)
	Q2	1048/6999	0.80 (0.70–0.90)	0.87 (0.77–0.99)	0.91 (0.80–1.04)	0.89 (0.78–1.01)
	Q3	898/6981	0.70 (0.61–0.81)	0.85 (0.75–0.97)	0.92 (0.79–1.08)	0.91 (0.78–1.06)
	Q4	741/6995	0.59 (0.50–0.68)	0.84 (0.72–0.97)	0.92 (0.76–1.10)	0.89 (0.75–1.07)
	Q5	521/6992	0.41 (0.35–0.47)	0.74 (0.64–0.87)	0.85 (0.70–1.04)	0.84 (0.69–1.03)
	P for trend		<.001	<.001	0.176	0.142
	Carotenoid	4456/34955	0.82 (0.78–0.87)	0.88 (0.83–0.93)	0.93 (0.88–0.98)	0.93 (0.88–0.98)
	Quintile					
	Q1	1118/6975	1.00 (Reference)	1.00 (Reference)	1.00 (Reference)	1.00 (Reference)
	Q2	911/6995	0.80 (0.69–0.93)	0.83 (0.74–0.95)	0.89 (0.78–1.01)	0.87 (0.76–0.99)
	Q3	851/6995	0.76 (0.66–0.87)	0.87 (0.76–0.99)	0.93 (0.82–1.06)	0.93 (0.82–1.05)
	Q4	827/6995	0.67 (0.58–0.78)	0.79 (0.69–0.90)	0.88 (0.78–1.00)	0.87 (0.77–0.98)
	Q5	749/6995	0.57 (0.49–0.65)	0.68 (0.59–0.78)	0.78 (0.68–0.90)	0.77 (0.67–0.88)
	P for trend		<.001	<.001	0.001	<.001
CVD mortality	Vitamin A	1368/34955	1.02 (0.96–1.08)	0.92 (0.83–1.00)	0.97 (0.89–1.04)	0.96 (0.89–1.04)
	Quintile					
	Q1	236/6983	1.00 (Reference)	1.00 (Reference)	1.00 (Reference)	1.00 (Reference)
	Q2	271/6995	1.06 (0.81–1.39)	0.86 (0.66–1.11)	0.92 (0.70–1.23)	0.93 (0.71–1.23)
	Q3	294/7007	1.29 (0.98–1.71)	0.93 (0.70–1.24)	1.05 (0.76–1.44)	1.04 (0.77–1.43)
	Q4	287/6982	1.15 (0.87–1.53)	0.74 (0.55–1.00)	0.86 (0.61–1.21)	0.86 (0.61–1.20)
	Q5	280/6988	1.12 (0.87–1.44)	0.75 (0.57–0.97)	0.91 (0.65–1.26)	0.90 (0.65–1.25)
	P for trend		0.485	0.012	0.439	0.387
	Vitamin E	1368/34955	0.71 (0.63–0.79)	0.81 (0.72–0.91)	0.86 (0.76–0.98)	0.86 (0.75–0.97)
	Quintile					
	Q1	416/7001	1.00 (Reference)	1.00 (Reference)	1.00 (Reference)	1.00 (Reference)
	Q2	309/6982	0.67 (0.54–0.83)	0.75 (0.61–0.92)	0.78 (0.63–0.97)	0.80 (0.65–0.99)
	Q3	273/7014	0.67 (0.52–0.86)	0.77 (0.62–0.97)	0.85 (0.66–1.09)	0.87 (0.67–1.12)
	Q4	223/6972	0.60 (0.48–0.74)	0.73 (0.59–0.92)	0.81 (0.61–1.09)	0.82 (0.61–1.10)
	Q5	147/6986	0.41 (0.30–0.55)	0.58 (0.43–0.78)	0.68 (0.49–0.94)	0.67 (0.48–0.92)
	P for trend		<.001	0.001	0.064	0.043
	Vitamin C	1368/34955	0.91 (0.85–0.98)	0.88 (0.80–0.97)	0.94 (0.86–1.03)	0.95 (0.86–1.03)
	Quintile					
	Q1	249/6987	1.00 (Reference)	1.00 (Reference)	1.00 (Reference)	1.00 (Reference)
	Q2	277/6993	1.13 (0.90–1.42)	0.94 (0.74–1.19)	0.99 (0.79–1.25)	0.98 (0.78–1.24)
	Q3	286/6995	1.09 (0.88–1.37)	0.80 (0.64–0.98)	0.91 (0.73–1.12)	0.90 (0.73–1.11)
	Q4	311/6992	1.17 (0.94–1.46)	0.78 (0.63–0.98)	0.89 (0.71–1.12)	0.88 (0.70–1.10)
	Q5	245/6988	0.88 (0.70–1.09)	0.72 (0.58–0.90)	0.87 (0.70–1.07)	0.86 (0.70–1.06)
	P for trend		0.118	0.003	0.140	0.146
	Zinc	1368/34955	0.82 (0.72–0.94)	0.98 (0.89–1.08)	1.03 (0.96–1.11)	1.03 (0.96–1.10)
	Quintile					
	Q1	330/6990	1.00 (Reference)	1.00 (Reference)	1.00 (Reference)	1.00 (Reference)
	Q2	309/6993	0.75 (0.60–0.94)	0.85 (0.66–1.10)	0.93 (0.71–1.21)	0.91 (0.70–1.18)
	Q3	274/7001	0.72 (0.57–0.90)	0.92 (0.73–1.14)	1.06 (0.83–1.37)	1.04 (0.80–1.33)
	Q4	239/6985	0.59 (0.46–0.77)	0.82 (0.63–1.08)	1.01 (0.74–1.39)	0.98 (0.72–1.35)
	Q5	216/6986	0.54 (0.40–0.71)	0.88 (0.66–1.18)	1.17 (0.80–1.71)	1.13 (0.77–1.66)
	P for trend		<.001	0.462	0.311	0.413
	Selenium	1368/34955	0.71 (0.64–0.79)	0.97 (0.88–1.07)	1.12 (0.99–1.28)	1.11 (0.97–1.27)
	Quintile					
	Q1	385/6988	1.00 (Reference)	1.00 (Reference)	1.00 (Reference)	1.00 (Reference)
	Q2	355/6999	0.83 (0.69–0.99)	0.93 (0.77–1.12)	1.03 (0.84–1.26)	1.02 (0.83–1.24)
	Q3	254/6981	0.62 (0.50–0.78)	0.80 (0.66–0.97)	0.94 (0.76–1.17)	0.92 (0.74–1.15)
	Q4	217/6995	0.54 (0.43–0.68)	0.85 (0.69–1.07)	1.08 (0.81–1.45)	1.06 (0.79–1.41)
	Q5	157/6992	0.41 (0.31–0.55)	0.92 (0.72–1.18)	1.30 (0.89–1.91)	1.27 (0.86–1.88)
	P for trend		<.001	0.355	0.189	0.244
	Carotenoid	1368/34955	0.85 (0.76–0.95)	0.92 (0.84–1.02)	0.97 (0.89–1.07)	0.97 (0.89–1.07)
	Quintile					
	Q1	318/6975	1.00 (Reference)	1.00 (Reference)	1.00 (Reference)	1.00 (Reference)
	Q2	296/6995	0.88 (0.68–1.14)	0.90 (0.71–1.14)	0.98 (0.77–1.24)	0.96 (0.76–1.22)
	Q3	280/6995	0.81 (0.64–1.04)	0.95 (0.74–1.22)	1.03 (0.81–1.32)	1.02 (0.79–1.30)
	Q4	242/6995	0.71 (0.56–0.91)	0.85 (0.68–1.08)	0.97 (0.78–1.22)	0.96 (0.77–1.20)
	Q5	232/6995	0.67 (0.51–0.89)	0.82 (0.63–1.07)	0.97 (0.74–1.27)	0.96 (0.73–1.25)
	P for trend		0.004	0.157	0.763	0.759
Cancer mortality	Vitamin A	1038/34955	1.04 (0.98–1.11)	0.96 (0.86–1.08)	1.00 (0.92–1.09)	1.00 (0.92–1.09)
	Quintile					
	Q1	192/6983	1.00 (Reference)	1.00 (Reference)	1.00 (Reference)	1.00 (Reference)
	Q2	180/6995	0.95 (0.64–1.40)	0.79 (0.53–1.16)	0.84 (0.57–1.23)	0.84 (0.57–1.23)
	Q3	211/7007	0.92 (0.70–1.22)	0.68 (0.51–0.91)	0.75 (0.56–1.01)	0.75 (0.56–1.01)
	Q4	212/6982	1.03 (0.78–1.35)	0.68 (0.51–0.91)	0.76 (0.56–1.04)	0.76 (0.56–1.04)
	Q5	243/6988	0.99 (0.75–1.30)	0.67 (0.51–0.89)	0.78 (0.58–1.04)	0.78 (0.59–1.04)
	*P* for trend		0.843	0.022	0.258	0.261
	Vitamin E	1038/34955	0.77 (0.68–0.86)	0.81 (0.72–0.91)	0.82 (0.71–0.94)	0.82 (0.71–0.94)
	Quintile					
	Q1	287/7001	1.00 (Reference)	1.00 (Reference)	1.00 (Reference)	1.00 (Reference)
	Q2	219/6982	0.91 (0.67–1.23)	0.96 (0.72–1.27)	0.97 (0.73–1.28)	0.96 (0.73–1.28)
	Q3	216/7014	0.95 (0.73–1.23)	0.99 (0.76–1.28)	1.02 (0.77–1.37)	1.02 (0.76–1.36)
	Q4	178/6972	0.69 (0.51–0.94)	0.73 (0.55–0.97)	0.75 (0.57–0.97)	0.74 (0.57–0.97)
	Q5	138/6986	0.55 (0.41–0.74)	0.63 (0.47–0.86)	0.67 (0.46–0.96)	0.67 (0.46–0.96)
	*P* for trend		<.001	<.001	0.007	0.007
	Vitamin C	1038/34955	0.90 (0.81–0.99)	0.86 (0.76–0.98)	0.91 (0.81–1.03)	0.91 (0.81–1.03)
	Quintile					
	Q1	217/6987	1.00 (Reference)	1.00 (Reference)	1.00 (Reference)	1.00 (Reference)
	Q2	222/6993	1.00 (0.72–1.37)	0.85 (0.62–1.15)	0.90 (0.66–1.23)	0.90 (0.65–1.23)
	Q3	207/6995	0.96 (0.73–1.27)	0.74 (0.57–0.95)	0.83 (0.64–1.09)	0.83 (0.64–1.09)
	Q4	195/6992	0.86 (0.64–1.16)	0.62 (0.47–0.82)	0.70 (0.52–0.94)	0.70 (0.53–0.93)
	Q5	197/6988	0.81 (0.60–1.10)	0.66 (0.49–0.89)	0.78 (0.59–1.04)	0.78 (0.58–1.04)
	P for trend		0.106	0.009	0.093	0.090
	Zinc	1038/34955	0.86 (0.75–0.99)	0.94 (0.84–1.06)	0.98 (0.90–1.06)	0.98 (0.90–1.06)
	Quintile					
	Q1	239/6990	1.00 (Reference)	1.00 (Reference)	1.00 (Reference)	1.00 (Reference)
	Q2	225/6993	0.87 (0.68–1.12)	0.91 (0.72–1.15)	0.93 (0.72–1.19)	0.93 (0.72–1.19)
	Q3	201/7001	0.70 (0.50–0.98)	0.78 (0.57–1.07)	0.82 (0.59–1.13)	0.81 (0.58–1.13)
	Q4	185/6985	0.64 (0.48–0.87)	0.74 (0.56–0.99)	0.78 (0.57–1.06)	0.78 (0.57–1.06)
	Q5	188/6986	0.59 (0.43–0.82)	0.74 (0.54–1.03)	0.80 (0.55–1.15)	0.79 (0.55–1.15)
	*P* for trend		<.001	0.056	0.206	0.205
	Selenium	1038/34955	0.76 (0.68–0.85)	0.91 (0.81–1.02)	0.94 (0.81–1.10)	0.94 (0.81–1.10)
	Quintile					
	Q1	264/6988	1.00 (Reference)	1.00 (Reference)	1.00 (Reference)	1.00 (Reference)
	Q2	231/6999	0.80 (0.64–1.01)	0.83 (0.66–1.05)	0.87 (0.68–1.11)	0.86 (0.68–1.11)
	Q3	215/6981	0.72 (0.52–1.01)	0.82 (0.59–1.13)	0.87 (0.61–1.23)	0.87 (0.61–1.24)
	Q4	181/6995	0.60 (0.46–0.78)	0.76 (0.57–1.02)	0.82 (0.58–1.15)	0.81 (0.58–1.14)
	Q5	147/6992	0.50 (0.37–0.69)	0.79 (0.57–1.09)	0.87 (0.57–1.33)	0.88 (0.58–1.33)
	P for trend		<.001	0.151	0.539	0.556
	Carotenoid	1038/34955	0.87 (0.78–0.96)	0.91 (0.82–1.01)	0.95 (0.85–1.05)	0.95 (0.85–1.05)
	Quintile					
	Q1	263/6975	1.00 (Reference)	1.00 (Reference)	1.00 (Reference)	1.00 (Reference)
	Q2	191/6995	0.86 (0.65–1.15)	0.87 (0.67–1.14)	0.91 (0.70–1.19)	0.91 (0.70–1.19)
	Q3	190/6995	0.90 (0.68–1.19)	0.99 (0.75–1.30)	1.05 (0.79–1.39)	1.05 (0.79–1.39)
	Q4	206/6995	0.75 (0.57–0.98)	0.83 (0.63–1.10)	0.91 (0.69–1.20)	0.91 (0.69–1.20)
	Q5	188/6995	0.61 (0.47–0.80)	0.69 (0.52–0.91)	0.77 (0.58–1.03)	0.77 (0.58–1.02)
	*P* for trend		<.001	0.006	0.057	0.056

HR: Hazard Ratio.

CI: Confidence Interval.

Model crude: Unadjusted.

Model1: Adjust for: gender, race, weight status, PIR, age.

Model2: Adjust for: gender, race, smoking, alcohol, weight status, PIR, PA, age, energy.

Model3: Adjust for: gender, race, smoking, alcohol, weight status, HBP, diabetes, PIR, PA, age, energy, CVD (unadjusted for CVD mortality), cancer (unadjusted for cancer mortality).

RCS analyses were consistent with Model 3 adjustments. For all-cause mortality, all dietary antioxidants showed a significant overall association (*p* < 0.05), with vitamins A and C demonstrating an ‘L’-shaped pattern and zinc a ‘U’-shaped pattern (nonlinear *p* < 0.05). For CVD mortality, vitamin E and selenium showed significant overall associations (*p* < 0.05), with selenium exhibiting a ‘U’-shaped association (nonlinear, *p* < 0.05). Finally, for cancer mortality, vitamins C and E showed a significant overall association (*p* < 0.05) but no nonlinear relationship (Figure 2A–C).

### Relationship between CDAI levels and mortality

Spearman’s analysis showed that the correlation values among the six dietary antioxidants ranged from 0.18 to 0.69, with vitamin E being the most closely related to the other antioxidants (Supplementary Figure 3). According to the WQS, vitamin A or C was the most significant contributor to both cause-specific and all-cause mortality reductions (Supplementary Figure 4A–C). RCS analysis found that an increase in CDAI was associated with a gradual decrease in the hazard ratio for both cause-specific and all-cause mortality but that excessively high levels of CDAI may increase the risk of all-cause mortality (Supplementary Figure 5A–C). K-M survival analyses indicated that Q4 and Q5 of CDAI showed higher survival compared with Q1 (Supplementary Figure 6A–C). Cox regression models assessed the relationship between CDAI levels and all-cause and cause-specific mortality (Supplementary Table 1). In Model 3, compared with Q1, participants in the highest quintile of the CDAI (Q5) had a significantly lower risk of all-cause mortality (HR: 0.82, 95% CI: 0.70–0.97) and cancer mortality (HR: 0.61, 95% CI: 0.45–0.84) (*P* for trend < 0.05).

### Subgroup analysis

Subgroup analyses were performed to assess the association between CDAI levels and mortality, with variables adjusted as in Model 3 (Table 3). Significant effect modification was observed by race for all-cause mortality and by gender for CVD mortality. Among participants under 65, the correlation between CDAI levels and cancer mortality was much greater.

**Table 3.** The modifications effect of subgroup on the association between total antioxidant intake levels and all-cause mortality as well as cause-specific mortality Table 3 long description.

Subgroups	*N* (%)	All-cause mortality		Cancer mortality		CVD mortality	
		HR (95%CI)	*P* interaction	HR (95%CI)	*P* interaction	HR (95%CI)	*P* interaction
Gender			0.105		0.241		0.005
Male	16626 (47.56)	0.99 (0.97∼1.01)		0.98 (0.93∼1.02)		1.00 (0.97∼1.03)	
Female	18329 (52.44)	0.97 (0.95∼1.00)		0.95 (0.89∼1.01)		0.97 (0.93∼1.02)	
Race			0.017		0.158		0.718
Mexican American	5483 (15.69)	0.95 (0.90∼1.01)		0.92 (0.82∼1.04)		0.99 (0.89∼1.09)	
Other Hispanic	2984 (8.54)	1.05 (0.98∼1.12)		1.12 (1.05∼1.19)		1.06 (0.91∼1.24)	
Non-Hispanic White	15812 (45.24)	0.98 (0.96∼1.00)		0.96 (0.92∼1.00)		0.98 (0.95∼1.01)	
Non-Hispanic Black	7463 (21.35)	1.03 (1.01∼1.06)		1.00 (0.94∼1.06)		1.03 (0.98∼1.08)	
Other race	3213 (9.19)	0.91 (0.84∼0.99)		0.99 (0.89∼1.11)		0.85 (0.74∼0.98)	
Alcohol use			0.239		0.446		0.063
Never/Light use	23979 (68.60)	0.98 (0.96∼1.00)		0.97 (0.93∼1.02)		0.98 (0.96∼1.01)	
Heavy use	10976 (31.40)	1.00 (0.97∼1.03)		0.95 (0.88∼1.03)		1.00 (0.94∼1.06)	
Age			0.794		0.008		0.841
<65 years	26250 (75.10)	0.99 (0.97∼1.02)		0.91 (0.85∼0.98)		0.99 (0.94∼1.04)	
>= 65 years	8705 (24.90)	0.98 (0.96∼1.00)		1.00 (0.96∼1.05)		0.99 (0.96∼1.02)	

HR: Hazard Ratio.

CI: Confidence Interval.

Model adjusted by age, gender, race, smoking status, alcohol use, weight status, hypertension, diabetes, family poverty income ratio, physical activity, CVD (unadjusted for CVD mortality), cancer (unadjusted for cancer mortality), energy.

## Discussion

Our study identified a significant association between increased intake of certain dietary antioxidants, particularly vitamin E, as well as higher CDAI levels, and reduced risk of cause-specific and all-cause mortality. However, causative factors underlying these associations remain unclear and warrant further investigation.

Our results echo a substantial body of observational work. Previous observational studies have consistently reported significant inverse associations between dietary antioxidants and all-cause and CVD mortality across various populations. For instance, analyses based on NHANES data indicated that increased intake of vitamin E and carotenoids were negatively associated with all-cause or CVD mortality.^(17)^ Dietary antioxidant intake (vitamin A, vitamin E and total carotenoid intake) was associated with reduced all-cause mortality in stroke patients.^(18)^ Adequate vitamin A intake limited to dietary sources is associated with reduced all-cause or CVD mortality.^(22)^ Moreover, higher dietary carotenoid intake and elevated blood concentrations of antioxidants were linked to lower mortality from CVD, cancer, and all causes.^(23)^ Our results extend these findings by demonstrating similar associations, particularly highlighting vitamin E’s pronounced relationship with reduced mortality risks. Previous studies have shown that supplementation with dietary antioxidants can reduce levels of oxidative stress and decrease inflammation.^(24–26)^

A credible biological explanation centres on oxidative stress — an excess of reactive oxygen or nitrogen species over antioxidant defences — which promotes both cardiovascular pathology and carcinogenesis.^(27)^ Oxidative stress harms health through two complementary routes:^(28)^ (i) direct molecular damage by highly reactive species that oxidise lipids, proteins, and DNA; and (ii) disruption of redox-sensitive signalling, whereby surplus hydrogen peroxide and related ROS destabilise normal cellular pathways.^(29,30)^ These twin mechanisms make it plausible that diets richer in antioxidants could attenuate disease processes and ultimately lower mortality risk.

While observational studies, including ours, consistently show protective associations between dietary antioxidants and reduced mortality, evidence from intervention trials has been less conclusive. A systematic review did not recommend high-dose supplementation of vitamins, minerals, and multivitamins for the prevention of CVD and cancer.^(31)^ Likewise, a 2024 pooled analysis of 390 124 adults in three prospective US cohorts found no survival benefit — and a 4% higher death rate during the first 12 years — among habitual multivitamin users.^(32)^ Such discrepancies likely arise because pharmacological-dose, isolated supplements differ fundamentally from the moderate, food-based antioxidants assessed in our study.^(33)^

Moreover, not all dietary antioxidants were found to be better at higher intakes, including vitamins A, C, and zinc, according to Cox regression results. This is consistent with the nonlinear ‘U’ shaped correlation in the RCS, suggesting that the protective effect of dietary antioxidants fluctuates with intake. Specifically, previous studies have demonstrated that zinc,^(34)^ selenium,^(35)^ and vitamin A^(36)^ possess both pro-inflammatory and antioxidant properties, and that excessive consumption of any of these nutrients can result in oxidative stress and inflammation. On the other hand, an overabundance of ROS scavenging may interfere with critical ROS-mediated intracellular signalling and metabolic functions. Such disruption of homeostasis may lead to increased mortality, thereby elucidating the “U”-shaped association observed in our findings.

Thus, at supraphysiological levels, some antioxidants may shift toward pro-oxidant behaviour. In contrast, antioxidants consumed as part of whole foods — embedded within complex matrices – are more likely to act synergistically and exhibit lower toxicity. These findings underscore the importance of achieving balanced, not maximal, antioxidant intake when formulating dietary guidelines and public health recommendations.

The six dietary antioxidants assessed in our study act through distinct yet complementary biological pathways. Vitamin A modulates transcriptional responses;^(37)^ vitamin E directly scavenges peroxyl radicals and limits lipid peroxidation;^(37)^ vitamin C donates electrons to neutralise free radicals at cell membranes;^(38)^ carotenoids quench singlet oxygen via their conjugated double-bond systems;^(39)^ selenium serves as a cofactor for redox-active selenoproteins that reduce hydroperoxides;^(40)^ and zinc stabilises protein thiols against radical attack.^(41)^ The convergence of these pathways helps explain why whole-food patterns — captured by the CDAI — appear more protective than any single nutrient.

Our findings align with this mechanistic framework. Participants with higher CDAI levels experienced significantly lower all-cause and cancer mortality, supporting the hypothesis that combined antioxidant exposure confers additive or synergistic protective effects. WQS regression further revealed that vitamin A or C had the greatest influence on mortality reduction, while vitamin E — despite being a strong predictor in single-nutrient models — received a lower weight due to its high correlation with other antioxidants. This finding underscores the importance of evaluating dietary patterns as a whole, rather than focusing on individual nutrients in isolation.

Notably, the inverse association between CDAI and cancer mortality was more pronounced among individuals under the age of 65, suggesting that younger adults may derive greater benefit from dietary antioxidant intake, possibly due to better redox homeostasis or more responsive antioxidant pathways. In contrast, the effect was weaker in older adults, who may face higher levels of oxidative stress that overwhelm dietary defences. In addition, there was an interaction for race in all-cause mortality, whereas there was an interaction for gender and a potential interaction for alcohol consumption in CVD mortality. According to a similar study, CDAI levels were negatively correlated with the risk of all-cause death only in men, not in women.^(42)^ In conclusion, our findings provide hypotheses for subsequent studies in specific populations.

This study draws on a large, nationally representative sample from the NHANES 2003–2018 cycles and uses a prospective cohort design, allowing for population-level inferences. By examining both individual dietary antioxidants and a combined index (CDAI), we were able to explore associations at multiple levels of exposure. The use of multiple statistical methods — including Cox models, restricted cubic splines, and weighted quantile sum regression — enabled us to assess both linear and nonlinear relationships. We also accounted for a wide range of covariates and conducted subgroup and interaction analyses to explore potential effect modification. These design features support the robustness of the observed associations.

Nonetheless, several limitations should be acknowledged. First, dietary data were collected at baseline using two 24-hour dietary recalls, which are subject to recall bias and may not accurately reflect long-term habitual intake patterns. Importantly, our analysis did not account for seasonal variations in food consumption. Because the availability and intake of fresh fruits and vegetables — primary sources of dietary antioxidants — fluctuate across seasons, reliance on short-term baseline recalls may capture seasonal peaks or troughs, potentially introducing intra-individual exposure misclassification. Temporal changes in dietary habits over the follow-up period also could not be captured. Second, the CDAI approach has inherent methodological limitations. The index is constructed based exclusively on a select few micronutrients and carotenoids, thus omitting other ubiquitous bioactive compounds — such as polyphenols and flavonoids — that contribute substantially to the total dietary antioxidant capacity. Furthermore, while the CDAI serves as a validated proxy for dietary antioxidant exposure, its correlation with physiological markers is complex. Previous epidemiological research demonstrates that while the CDAI inversely correlates with systemic inflammatory markers, such as tumour necrosis factor-alpha (TNF-α) and interleukin-1 beta (IL-1β), it does not significantly correlate with direct physiological biomarkers of oxidative stress, such as urinary F2-isoprostanes.^(43)^ Because our study lacked concurrent physiological biomarker data, the CDAI should be interpreted as an index of aggregate dietary exposure rather than a direct surrogate for internal physiological redox status. Third, the study did not include detailed information on dietary supplement use and prescription medications, which may have led to an underestimation of total antioxidant exposure. Finally, although we used prospective follow-up mortality data, the analytical design remains observational. Thus, residual confounding cannot be fully excluded, and causal inference cannot be established.

Priority should be given to future research that involves large-scale, carefully designed randomised controlled trials and long-term prospective intervention studies to directly evaluate the causal impact of dietary antioxidant intake on all-cause and cause-specific mortality. Such studies are critical for identifying optimal intake levels and population subgroups most likely to benefit, ultimately informing evidence-based dietary guidelines and public health strategies.

## Conclusion

Higher intakes of vitamin E and CDAI were associated with lower all-cause and cancer mortality in a US population. U-shaped associations for some nutrients suggest potential harm from both low and high intake. These findings highlight the value of balanced, food-based antioxidant consumption. Causal relationships remain uncertain and warrant further study.

## Supporting information

10.1017/jns.2026.10117.sm001 Liu et al. supplementary material 1 Liu et al. supplementary material

10.1017/jns.2026.10117.sm002 Liu et al. supplementary material 2 Liu et al. supplementary material

10.1017/jns.2026.10117.sm003 Liu et al. supplementary material 3 Liu et al. supplementary material

10.1017/jns.2026.10117.sm004 Liu et al. supplementary material 4 Liu et al. supplementary material

10.1017/jns.2026.10117.sm005 Liu et al. supplementary material 5 Liu et al. supplementary material

10.1017/jns.2026.10117.sm006 Liu et al. supplementary material 6 Liu et al. supplementary material

10.1017/jns.2026.10117.sm007 Liu et al. supplementary material 7 Liu et al. supplementary material

## Data Availability

The NHANES dataset utilised in this study is accessible through the National Center for Health Statistics (https://wwwn.cdc.gov/nchs/nhanes/default.aspx).

## Supplementary material

The supplementary material for this article can be found at https://doi.org/10.1017/jns.2026.10117.

## Figure 1. Long description

The flowchart begins with 80,312 participants from NHANES 2003-2018. Exclusion criteria are applied, removing participants under 20 years old (35,522), those missing dietary data of two days (9,757), and participants without mortality data (57). This leaves 34,976 participants. Further exclusions for other missing covariate data (21) result in a final inclusion of 34,955 participants.

Navigate back to Figure 1..

## Table 1. Long description

The table presents baseline characteristics of participants categorized by CDAI quintile. It includes data on 34,955 participants with a median age of 47.00 years, 52.3% of whom are female and 68.05% are non-Hispanic White. The table is divided into five quintiles (Q1 to Q5) with 6991 participants each. Key variables include age, energy intake, gender distribution, race, smoking status, alcohol use, weight status, hypertension, diabetes, family poverty income ratio, physical activity, cardiovascular disease (CVD), and cancer. Notable trends include higher CDAI levels (Q5) being more frequent among younger, male participants who are more physically active, have higher incomes, and greater energy intake. These participants are also more likely to be current smokers, consume never/light amounts of alcohol, and have no baseline history of hypertension, diabetes, CVD, or cancer.

Navigate back to Table 1..

## Figure 2. Long description

The image contains three sets of graphs labeled (a), (b), and (c), each showing the association of dietary antioxidants with different types of mortality risk. Each set includes four individual graphs. The x-axes of the graphs represent the log-transformed intake of various dietary antioxidants in milligrams, while the y-axes represent the hazard ratio. The graphs use restricted cubic spline curves to illustrate the relationships. In set (a), the graphs show the all-cause mortality risk associated with Vitamin A, Vitamin C, Vitamin E, Selenium, Zinc, and Carotenoid intake. Set (b) displays the CVD mortality risk for the same antioxidants, and set (c) shows the cancer mortality risk. Each graph includes p-values for overall and nonlinear associations, indicating the statistical significance of the observed trends. The blue lines represent the hazard ratios, with shaded areas indicating confidence intervals. The red dashed lines represent a hazard ratio of 1, serving as a reference point. The graphs reveal various trends, such as a decrease in hazard ratio with increasing intake of certain antioxidants, suggesting potential protective effects against mortality.

Navigate back to Figure 2..

## Table 2. Long description

The table presents data on the association between dietary intake of antioxidants and mortality rates, including all-cause mortality and cause-specific mortality. It consists of 13 rows and 10 columns. The columns are labeled with variables such as Outcome, Sample Size, Events, Hazard Ratio (HR) with 95% Confidence Intervals (CI) for Model 1, Model 2, and Model 3, and P for trend. The rows are categorized by different outcomes: All-cause mortality, Cardiovascular Disease (CVD) mortality, and Cancer mortality. Each row provides specific data points for these categories, showing the relationship between dietary antioxidant intake and mortality risks. Notable trends include significant associations between higher quintiles of vitamin E and carotenoids with lower all-cause mortality, and protective associations of vitamin E with CVD mortality.

Navigate back to Table 2..

## Table 3. Long description

The table presents subgroup analyses to assess the association between CDAI levels and mortality, with adjustments made as in Model 3. The table includes data on all-cause mortality, cancer mortality, and cardiovascular disease (CVD) mortality across different subgroups such as gender, race, alcohol use, and age. The table has 12 rows and 7 columns. The columns are labeled Subgroups, N (percentage), All-cause mortality HR (95% CI), P interaction, Cancer mortality HR (95% CI), P interaction, and CVD mortality HR (95% CI), P interaction. The subgroups include gender (Male, Female), race (Mexican American, Other Hispanic, Non-Hispanic White, Non-Hispanic Black, Other race), alcohol use (Never/Light use, Heavy use), and age (<65 years, >= 65 years). Notable trends include significant effect modification by race for all-cause mortality and by gender for CVD mortality. Among participants under 65, the correlation between CDAI levels and cancer mortality was much greater.

Navigate back to Table 3..
