# Supplementary figures and images for: Independent and combined associations of dietary antioxidant exposure with all-cause and cause-specific mortality in the general population
Source: J Nutr Sci. 2026 Jul 7;15:e53. doi: 10.1017/jns.2026.10117 (PMC13369252; doi:10.1017/jns.2026.10117)

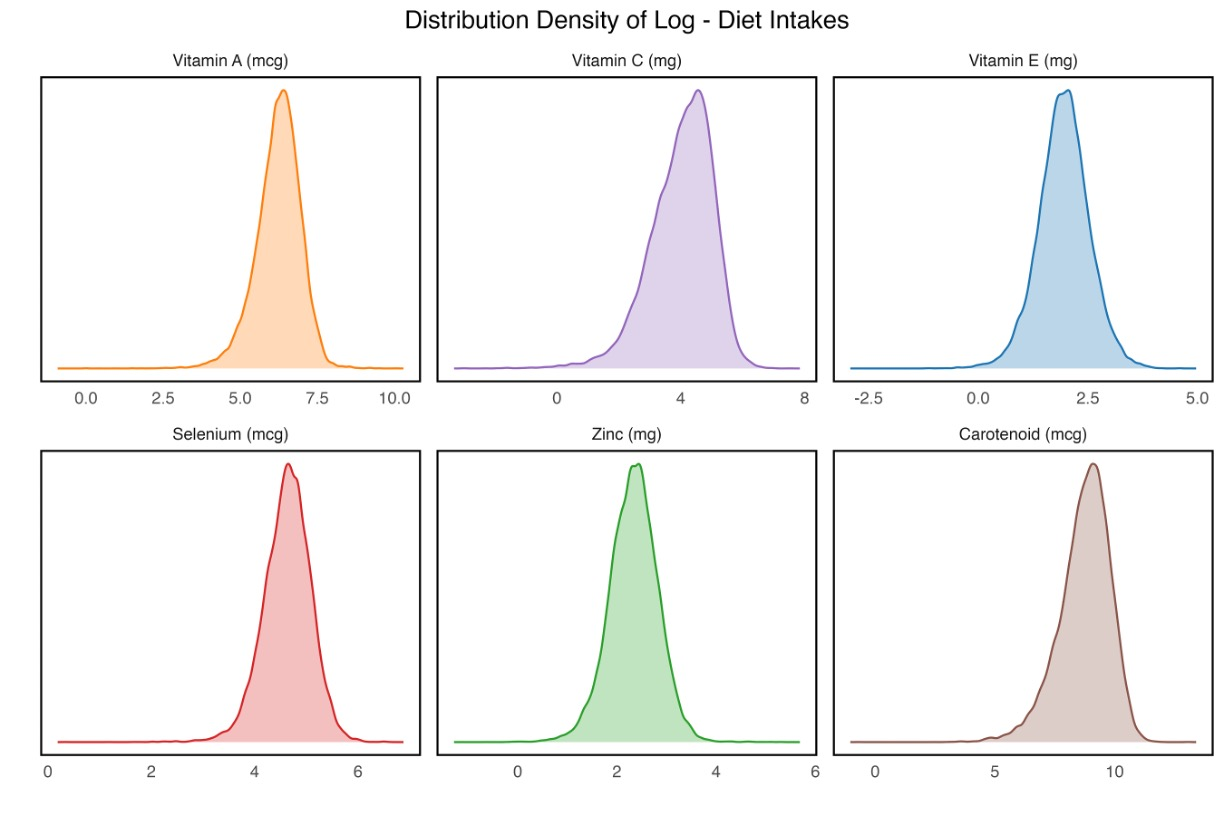

Supplement: Liu et al. supplementary material 1 — Liu et al. supplementary material [file S2048679026101177sup001.tiff]

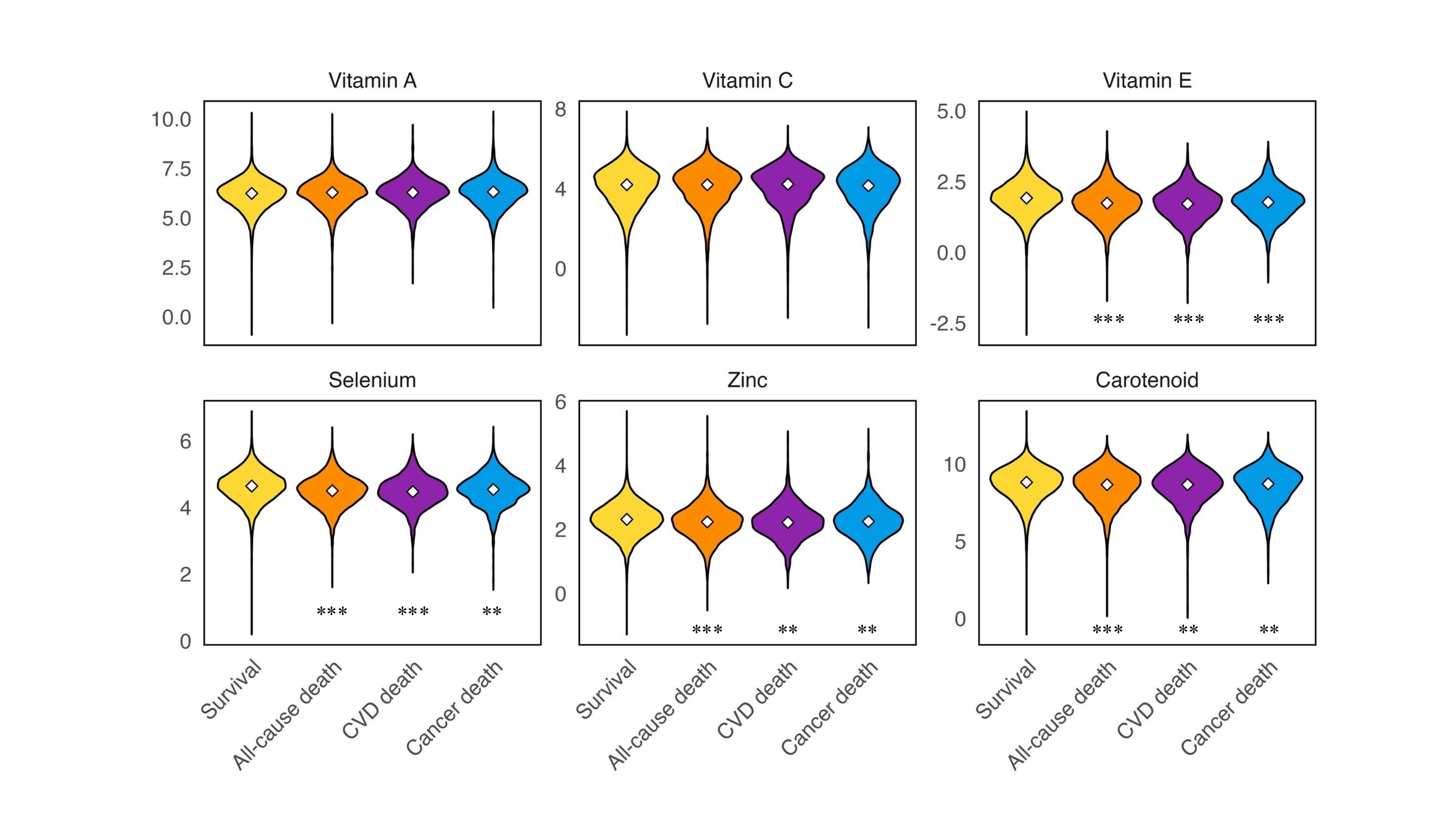

Supplement: Liu et al. supplementary material 2 — Liu et al. supplementary material [file S2048679026101177sup002.tiff]

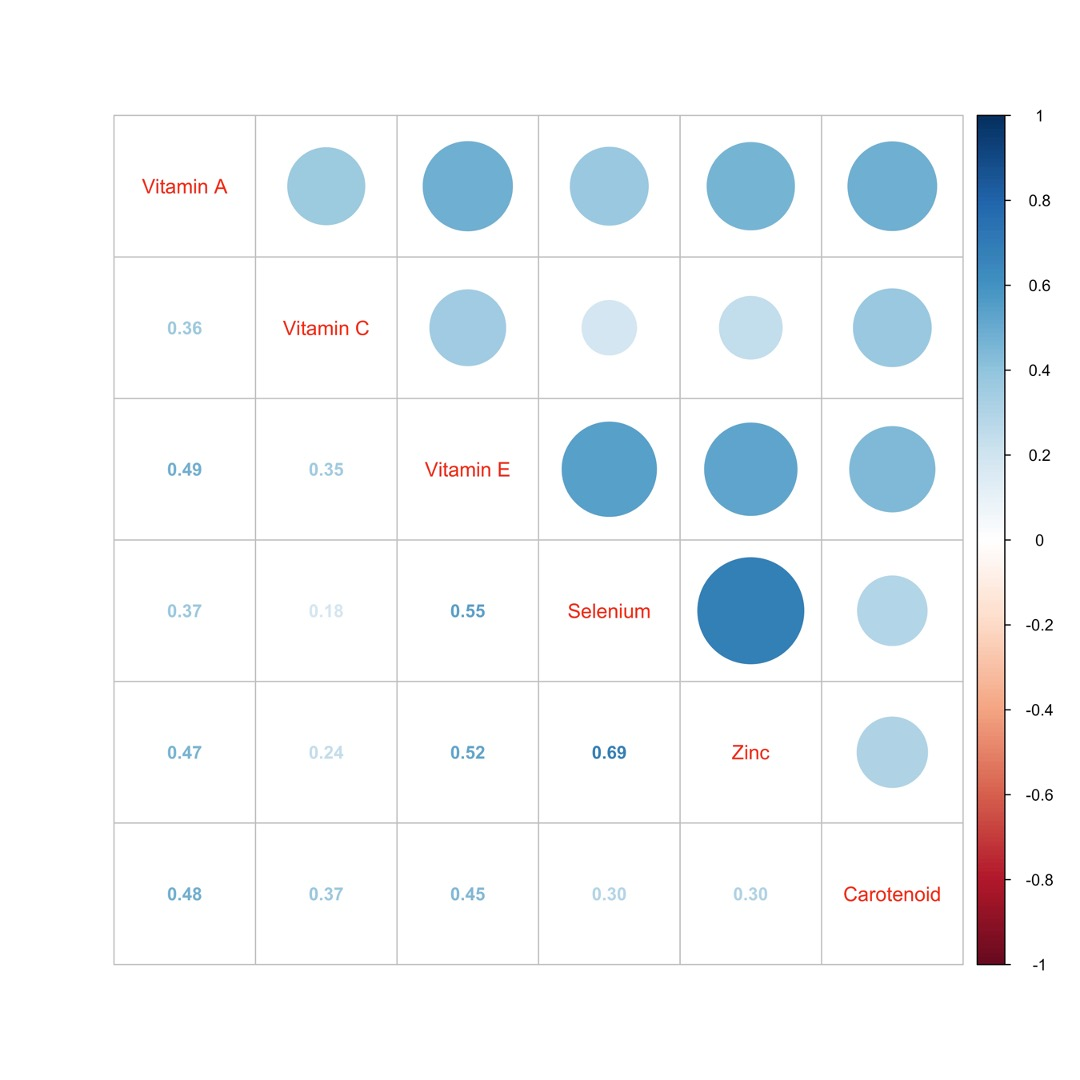

Supplement: Liu et al. supplementary material 3 — Liu et al. supplementary material [file S2048679026101177sup003.tiff]

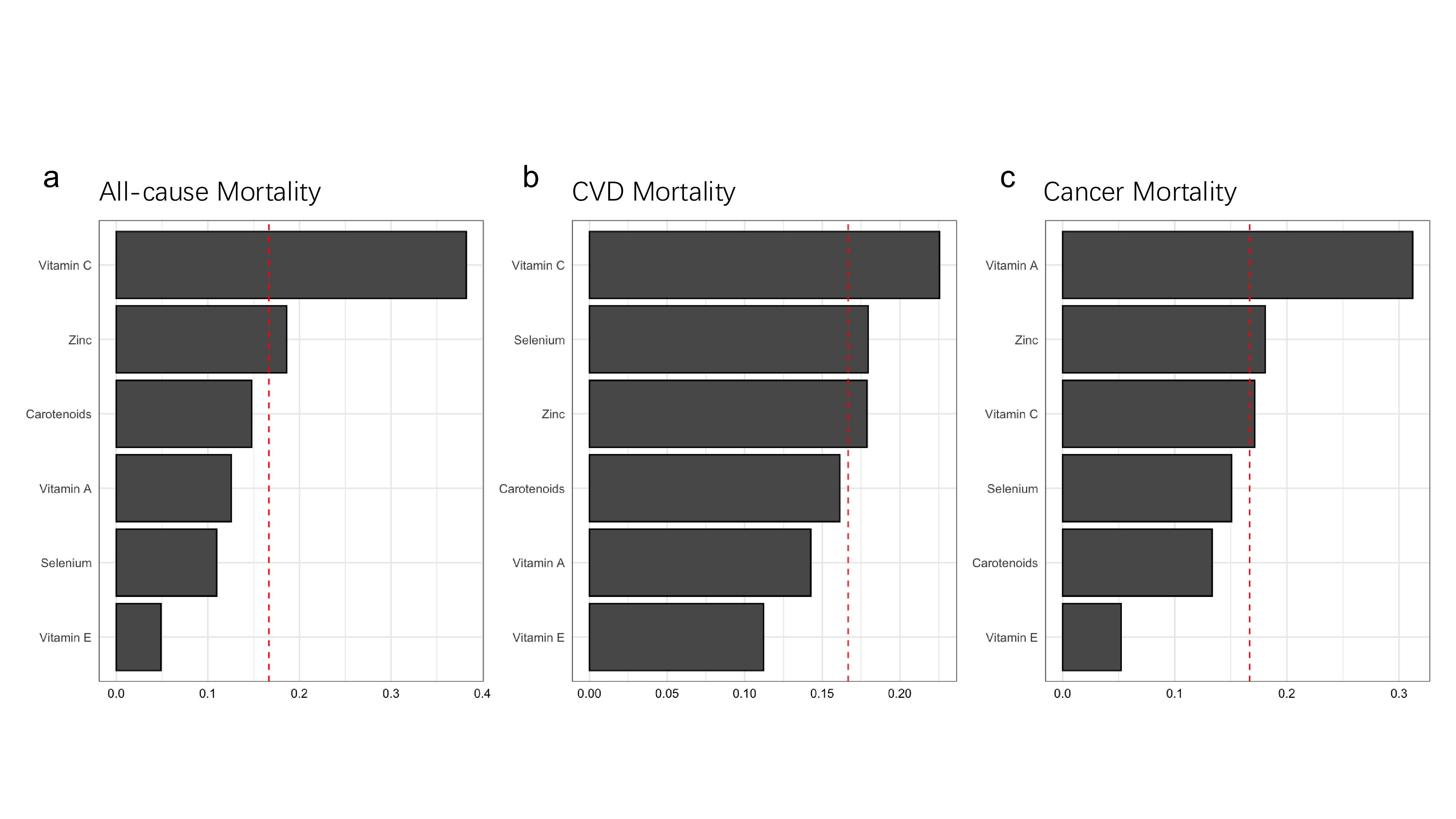

Supplement: Liu et al. supplementary material 4 — Liu et al. supplementary material [file S2048679026101177sup004.tiff]

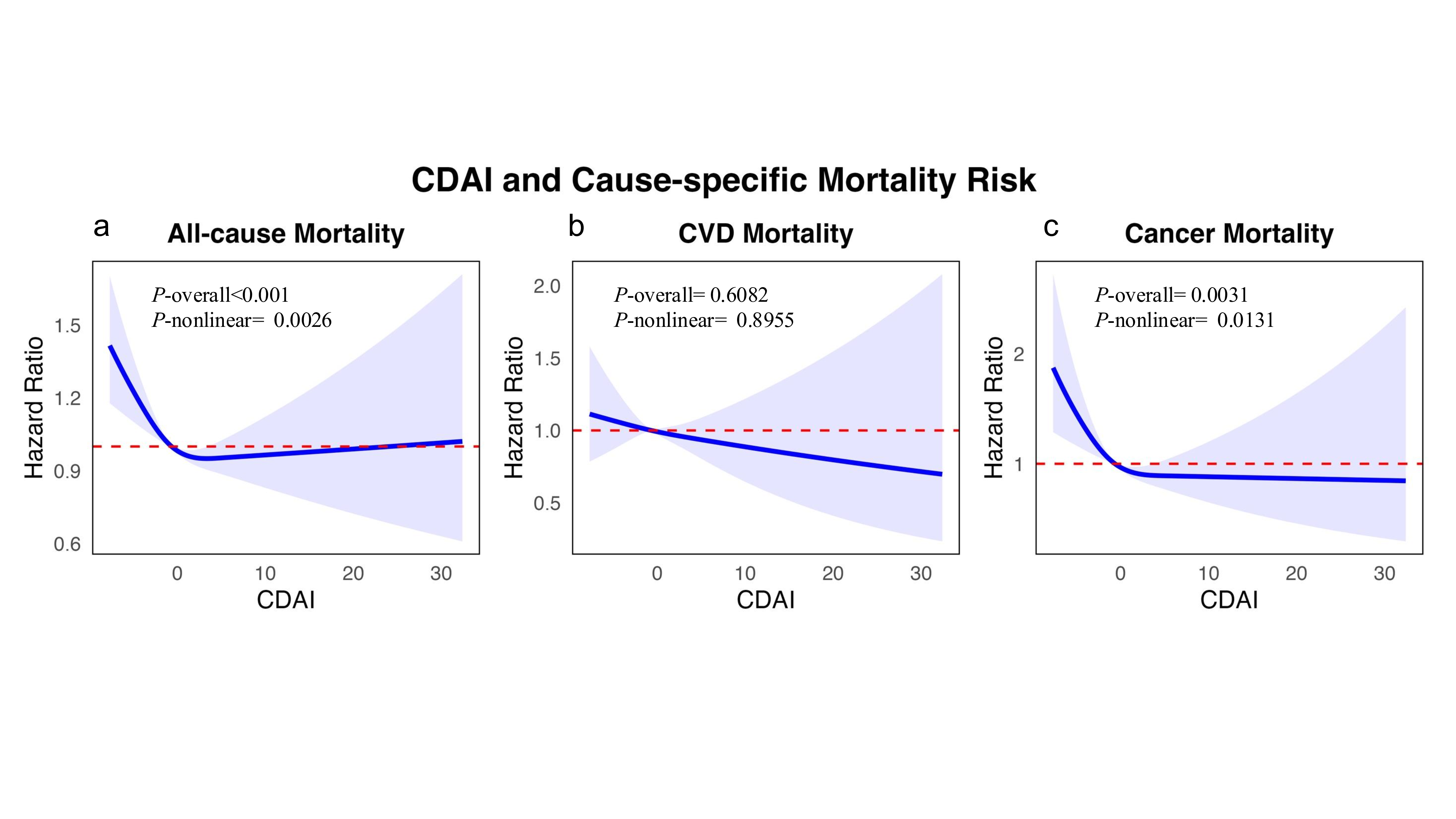

Supplement: Liu et al. supplementary material 5 — Liu et al. supplementary material [file S2048679026101177sup005.tiff]

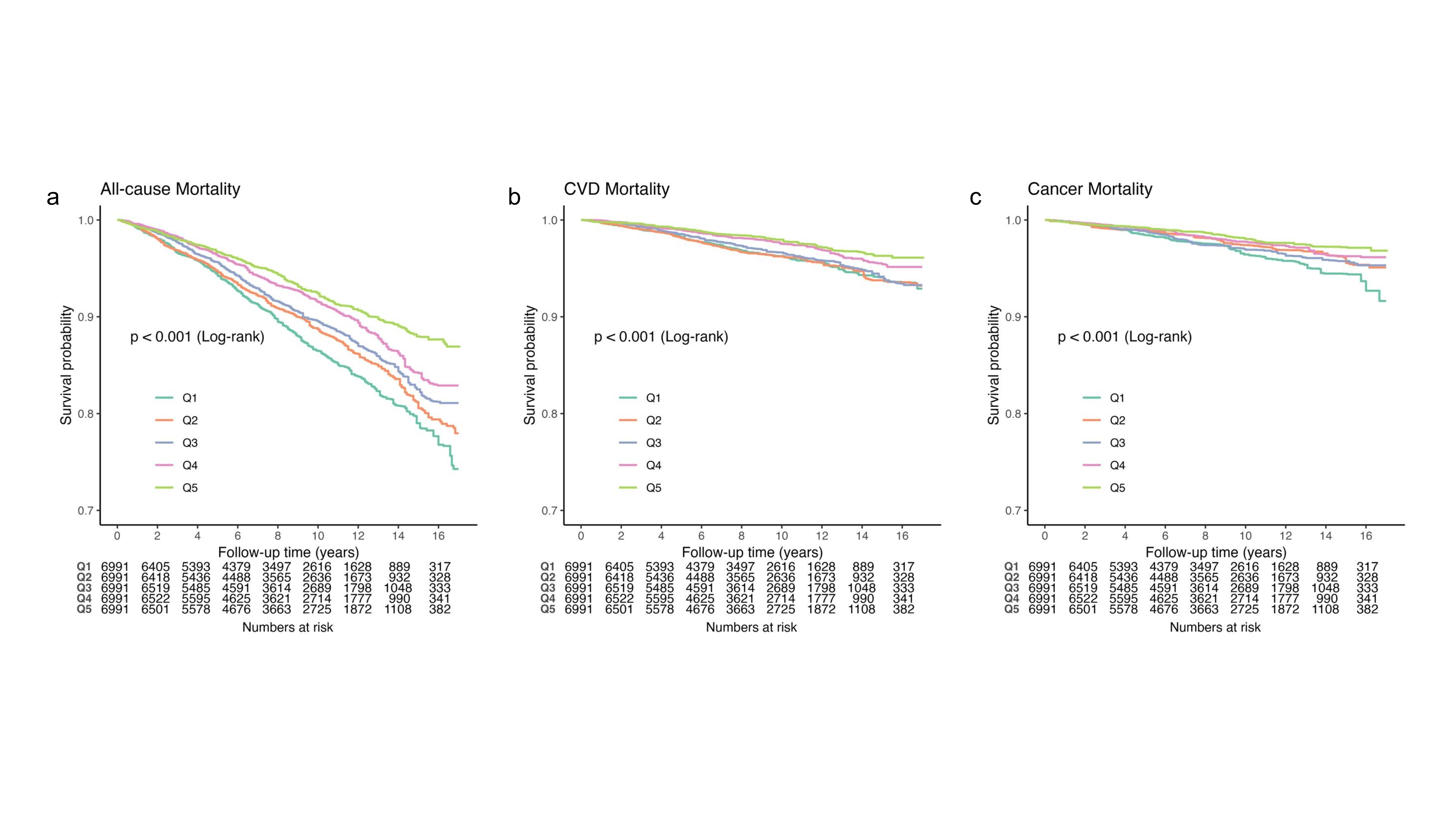

Supplement: Liu et al. supplementary material 6 — Liu et al. supplementary material [file S2048679026101177sup006.tiff]
