# Supplementary material for: Independent and combined associations of dietary antioxidant exposure with all-cause and cause-specific mortality in the general population
Source: J Nutr Sci. 2026 Jul 7;15:e53. doi: 10.1017/jns.2026.10117 (PMC13369252; doi:10.1017/jns.2026.10117)
Supplement: Liu et al. supplementary material 7 — Liu et al. supplementary material [file S2048679026101177sup007.docx]

Supplementary Table 1. The association between total antioxidant intake levels and all-cause mortality and mortality from specific diseases.

| Outcomes | Death/person | Model crude | Model 1 | Model 2 | Model 3 |
| --- | --- | --- | --- | --- | --- |
|  |  | HR (95%CI) | HR (95%CI) | HR (95%CI) | HR (95%CI) |
| All-cause mortality |  |  |  |  |  |
| CDAI, continuous | 4456/34955 | 0.80 (0.76 - 0.85) | 0.88 (0.83 - 0.94) | 0.95 (0.89 - 1.01) | 0.94 (0.88 - 1.00) |
| CDAI, Quintile |  |  |  |  |  |
| Q1 | 1097/6991 | 1.00 (Reference) | 1.00 (Reference) | 1.00 (Reference) | 1.00 (Reference) |
| Q2 | 1021/6991 | 0.87 (0.76 - 1.00) | 0.87 (0.76 - 1.00) | 0.93 (0.81 - 1.07) | 0.91 (0.79 - 1.04) |
| Q3 | 899/6991 | 0.78 (0.69 - 0.89) | 0.84 (0.73 - 0.96) | 0.95 (0.82 - 1.10) | 0.92 (0.80 - 1.07) |
| Q4 | 782/6991 | 0.65 (0.56 - 0.77) | 0.73 (0.63 - 0.84) | 0.82 (0.69 - 0.97) | 0.81 (0.68 - 0.96) |
| Q5 | 657/6991 | 0.54 (0.46 - 0.64) | 0.69 (0.59 - 0.81) | 0.83 (0.70 - 0.99) | 0.82 (0.70 - 0.97) |
| P for trend |  | <.001 | <.001 | 0.019 | 0.015 |
| CVD mortality |  |  |  |  |  |
| CDAI, continuous | 1368/34955 | 0.77 (0.70 - 0.85) | 0.87 (0.79 - 0.96) | 0.96 (0.86 - 1.07) | 0.95 (0.86 - 1.06) |
| CDAI, Quintile |  |  |  |  |  |
| Q1 | 319/6991 | 1.00 (Reference) | 1.00 (Reference) | 1.00 (Reference) | 1.00 (Reference) |
| Q2 | 337/6991 | 1.01 (0.81 - 1.25) | 1.01 (0.81 - 1.26) | 1.11 (0.89 - 1.38) | 1.09 (0.87 - 1.36) |
| Q3 | 290/6991 | 0.91 (0.73 - 1.12) | 0.98 (0.80 - 1.22) | 1.17 (0.92 - 1.49) | 1.16 (0.91 - 1.47) |
| Q4 | 236/6991 | 0.66 (0.52 - 0.84) | 0.76 (0.61 - 0.95) | 0.94 (0.71 - 1.24) | 0.93 (0.70 - 1.23) |
| Q5 | 186/6991 | 0.56 (0.43 - 0.73) | 0.79 (0.60 - 1.02) | 1.06 (0.77 - 1.45) | 1.03 (0.75 - 1.41) |
| P for trend |  | <.001 | 0.010 | 0.875 | 0.774 |
| Cancer mortality |  |  |  |  |  |
| CDAI, continuous | 1038/34955 | 0.82 (0.73 - 0.91) | 0.85 (0.76 - 0.96) | 0.88 (0.76 - 1.01) | 0.88 (0.76 - 1.01) |
| CDAI, Quintile |  |  |  |  |  |
| Q1 | 256/6991 | 1.00 (Reference) | 1.00 (Reference) | 1.00 (Reference) | 1.00 (Reference) |
| Q2 | 203/6991 | 0.72 (0.54 - 0.97) | 0.69 (0.52 - 0.91) | 0.71 (0.54 - 0.93) | 0.71 (0.54 - 0.94) |
| Q3 | 214/6991 | 0.81 (0.61 - 1.06) | 0.81 (0.61 - 1.06) | 0.85 (0.64 - 1.13) | 0.85 (0.64 - 1.13) |
| Q4 | 202/6991 | 0.63 (0.48 - 0.82) | 0.62 (0.48 - 0.81) | 0.65 (0.47 - 0.89) | 0.65 (0.47 - 0.89) |
| Q5 | 163/6991 | 0.52 (0.38 - 0.71) | 0.58 (0.43 - 0.77) | 0.61 (0.45 - 0.83) | 0.61 (0.45 - 0.84) |
| P for trend |  | <.001 | <.001 | 0.009 | 0.009 |

HR: Hazard Ratio; CI: Confidence Interval. Model adjusted by age, gender, race, smoking status, alcohol use, weight status, hypertension, diabetes, family poverty income ratio, physical activity, CVD (unadjusted for CVD mortality), cancer (unadjusted for cancer mortality), energy.
